# Ambient AI scribes in rheumatology: early real-world clinician and patient experience

**DOI:** 10.1016/j.ero.2025.12.002

**Published:** 2025-12-19

**Authors:** Johannes Knitza, Peer Aries

**Affiliations:** 1 Institute for Digital Medicine, University Hospital Marburg, Philipps-Universität Marburg, Marburg, Germany; 2 Department of Rheumatology, Immunologikum, Hamburg, Germany

Dear Editor,

Artificial intelligence (AI) is reshaping rheumatology, including the traditional physician–patient relationship that is increasingly becoming a triad with AI systems [1]. Clinicians anticipate that AI will reduce documentation and administrative workload [2]. Ambient AI scribes, powered by large language models (LLMs) that convert physician–patient conversations into structured clinical notes, show considerable promise. A large-scale evaluation involving over 2.5 million uses by the Permanente Medical Group demonstrated substantial time savings, highly positive feedback from physicians, and neutral to positive responses from patients [3]. A recent rapid review on ambient AI scribe systems corroborated these findings, including studies from oncology, dermatology, and primary care. However, it also highlighted that the overall evidence base remains limited and the methodologies employed are heterogeneous [4]. Notably, real-world data in rheumatology are still lacking.

The aim of this study was to evaluate the LLM-based medical scribe (nixiai.ai; Nixi AI) used during routine rheumatology consultations. Patients were consecutively recruited at a large German rheumatology outpatient service in August 2025. Evaluation questionnaires (Web-based REDCap [Research Electronic Data Capture]; Vanderbilt University) were based on 2 AI scribe evaluation frameworks [3,5]. The Ethics Committee of Philipps University Marburg, Germany, confirmed exemption owing to the anonymous and noninterventional study design (25-215 ANZ). We collected feedback from the treating rheumatologist on the perceived time required per case and on documentation errors, as well as from patients on acceptance and perceived consultation quality. Patient feedback included questions on prior use of LLMs for health-related purposes, general acceptance of LLMs in healthcare, and 4 items based on a previous study [3], assessing the ambient scribe experience using a 5-point Likert scale (1 = totally disagree, 5 = totally agree). Errors were evaluated following the framework proposed by Asgari et al [5], where errors that could potentially alter patient diagnosis or management if uncorrected were classified as major.

The rheumatologist self-reported that the AI scribe reduced consultation time in 72 of 88 cases (82%), increased it in 3 cases (3%), and had no effect in 13 cases (15%) (Fig, A). Errors were reported in 17 of 88 (19%) encounters, consisting of misspelled medication names and incorrect sex (female/male) detection. The latter reflected an automated classification function unique to this system rather than clinician misdocumentation. All errors were rated as minor.

**Figure:**
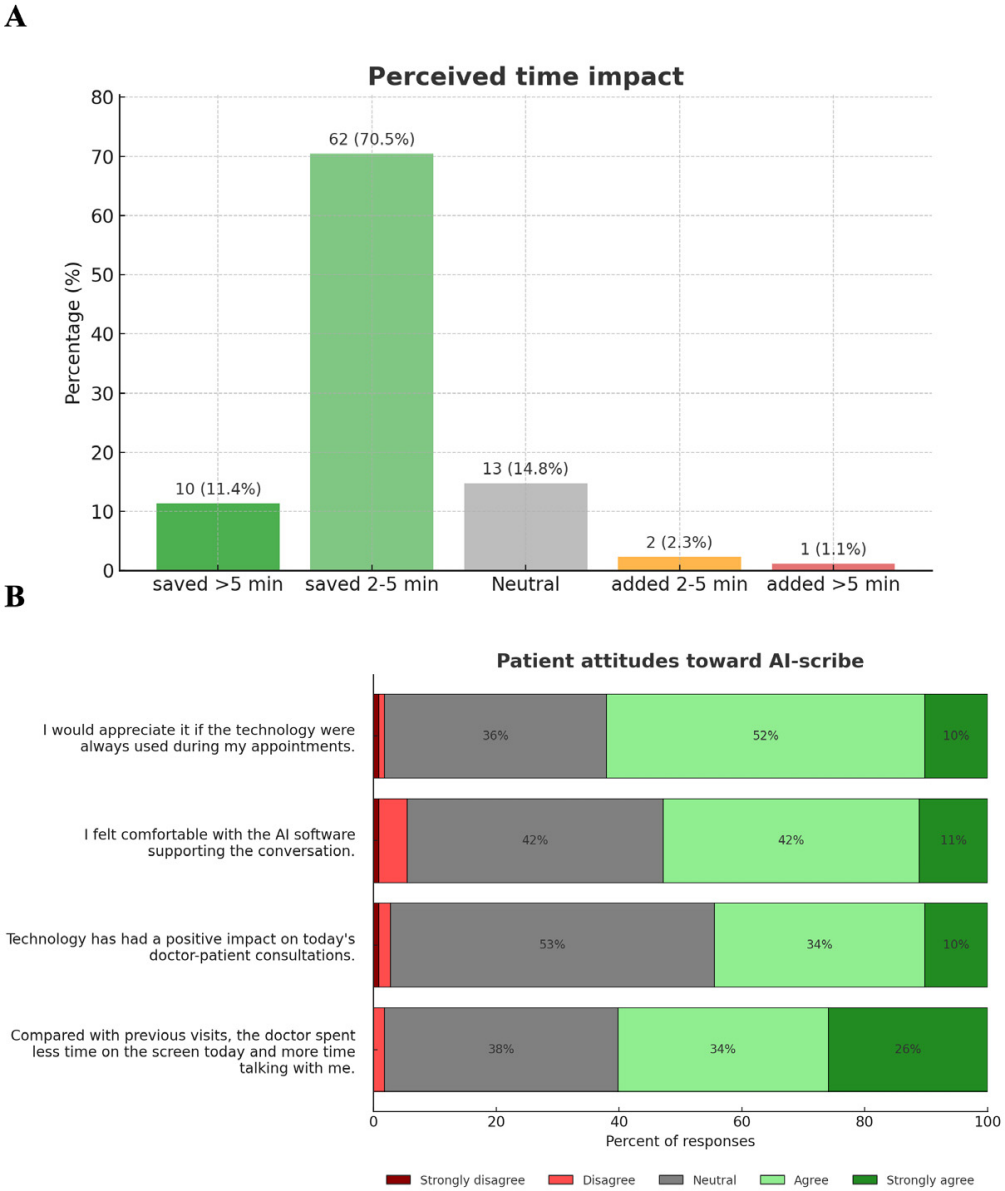
A, Rheumatologist perceived the time impact of the ambient AI scribe. B, Patient attitudes towards ambient AI scribe. AI, artificial intelligence.

Questionnaires were completed by 108 patients (64.8% female, mean age [SD] 59 years [15]). No patients declined participation, highlighting acceptance and reducing the likelihood of selection bias. Twelve encounters were first visits, and 96 were follow-ups. Thirty-six patients (33%) had previously used AI tools for health-related inquiries. Attitudes towards AI in healthcare were neutral in 32 (30%) patients, positive in 61 (56%), and very positive in 15 (14%). Most patients indicated that they would welcome the use of the scribe in future visits, felt comfortable with AI support during the conversation, and perceived that their physician spent less time looking at the screen and more time engaging with them (Fig, B).

These preliminary findings align with prior evaluations in nonrheumatology settings, demonstrating meaningful time savings, low error rates, and high levels of acceptance among both patients and physicians [3,4]. Furthermore, LLMs hold promise for enhancing face-to-face consultations by providing on-demand, chatbot-based support, thereby making medical information more comprehensive and accessible [6].

Limitations include the single-centre and single-rheumatologist design, modest sample size, self-reported nature of perceived time impact, and the short observation period without longitudinal follow-up. Interoperability remains a key practical consideration for implementation. Currently, outputs frequently necessitate manual copy–paste transfer into the medical report and subsequently into a structured electronic health record format, as observed in this study. Future studies should examine longitudinal effects, including risks such as automation bias and overreliance, and assess how documentation completeness and accuracy evolve over time. Harmonised evaluation frameworks, including recorded standardised conversations and safety metrics, are needed to enable comparison across settings and to support safe real-world implementation.

## Editor disclosure

The peer review process did not involve Editorial Board Member Johannes Knitza, and the editorial decision-making was led by editors who were not involved in the creation of this manuscript.

## CRediT authorship contribution statement

**Johannes Knitza:** Writing – review & editing, Writing – original draft, Visualization, Supervision, Resources, Project administration, Methodology, Investigation, Formal analysis, Data curation, Conceptualization. **Peer Aries:** Writing – review & editing, Supervision, Software, Resources, Project administration, Methodology, Investigation, Data curation, Conceptualization.

## Competing interests

JK is an associate editor of ERO. PA reports no conflicts of interest.
